# Age-related dysregulation of intestinal epithelium fucosylation is linked to an increased risk of colon cancer

**DOI:** 10.1172/jci.insight.167676

**Published:** 2024-01-30

**Authors:** Zhihan Wang, Pan Gao, Kai Guo, Grace Schirrick, Jappreet Singh Gill, Jett Weis, Abby Lund Da Costa, Mansib Rahman, Het Mehta, Julia Fleecs, Shilpi Jain, Trishna Debnath, Junguk Hur, Nadeem Khan, Robert Sticca, Holly M. Brown-Borg, Donald A. Jurivich, Ramkumar Mathur

**Affiliations:** 1 Department of Geriatrics, School of Medicine and Health Sciences, University of North Dakota, Grand Forks, North Dakota, USA.; 2 State Key Laboratory of Biotherapy and Cancer Center, West China Hospital, West China School of Basic Medical Sciences & Forensic Medicine, and Collaborative Innovation Center for Biotherapy, Sichuan University, Chengdu, China.; 3 Department of Urology, Frontier Science Center for Immunology and Metabolism, and Medical Research Institute, Zhongnan Hospital of Wuhan University, Wuhan University, Wuhan, China.; 4 Department of Biomedical Sciences, School of Medicine and Health Sciences, University of North Dakota, Grand Forks, North Dakota, USA.; 5 Department of Neurology, University of Michigan School of Medicine, Ann Arbor, Michigan, USA.; 6 Department of Oral Biology, College of Dentistry, University of Florida, Gainesville, Florida, USA.; 7 Department of Surgery, School of Medicine and Health Sciences, University of North Dakota, Grand Forks, North Dakota, USA.

**Keywords:** Aging, Microbiology, Cellular senescence, Colorectal cancer, Drug therapy

## Abstract

Colon cancer affects people of all ages. However, its frequency, as well as the related morbidity and mortality, are high among older adults. The complex physiological changes in the aging gut substantially limit the development of cancer therapies. Here, we identify a potentially unique intestinal microenvironment that is linked with an increased risk of colon cancer in older adults. Our findings show that aging markedly influenced persistent fucosylation of the apical surfaces of intestinal epithelial cells, which resulted in a favorable environment for tumor growth. Furthermore, our findings shed light on the importance of the host-commensal interaction, which facilitates the dysregulation of fucosylation and promotes tumor growth as people get older. We analyzed colonic microbial populations at the species level to find changes associated with aging that could contribute to the development of colon cancer. Analysis of single-cell RNA-sequencing data from previous publications identified distinct epithelial cell subtypes involved in dysregulated fucosylation in older adults. Overall, our study provides compelling evidence that excessive fucosylation is associated with the development of colon cancer, that age-related changes increase vulnerability to colon cancer, and that a dysbiosis in microbial diversity and metabolic changes in the homeostasis of older mice dysregulate fucosylation levels with age.

## Abstract 2

Excessive gut epithelial cell fucosylation increases the risk of colon cancer in the elderly.

## Graphical abstract

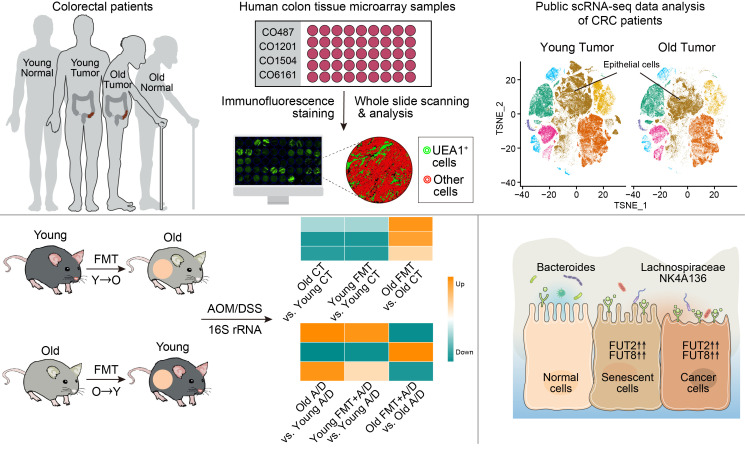

## Introduction

Colorectal cancer ranks as the third most notable contributor to cancer-related mortality in the United States and represents a substantial public health issue worldwide (1–4). Remarkably, older adults have a higher mortality rate of colorectal cancer compared with young individuals, highlighting the crucial role of age-related factors in fostering tumor development among older adults (5, 6). In recent years, studies have focused on deciphering the intricate relationship between the gut environment and the increasing incidence of colon cancer. Elucidating the complex gut microenvironment is essential to develop preventive and therapeutic strategies for colon cancer.

The gut epithelial cells have diverse functions, serving as a barrier between immune cells and commensals and pathogens under a homeostatic condition. However, as people age, dysregulation of the gut environment creates a favorable environment for adenomatous precancerous polyps to proceed to malignancy (7, 8). A wide range of microbiota show a symbiotic relationship and have been shown to support healthy aging (9). Conversely, certain microbial varieties are linked to dysbiosis and produce metabolic compounds that contribute to degenerative aging and the risk of colon cancer (10–12). A better understanding of age-related factors and the microenvironment within the colon holds promise for establishing a mutually beneficial association with the gut microbiota in the progression and management of colon cancer among older individuals.

Central to understanding the relationship between the gut microbiome and colonic integrity is the process of fucosylation, which maintains the intestinal lining and gut homeostasis. Fucosylation is a posttranslational modification of glycans, proteins, and lipids that results in the transfer of fucose residues to oligosaccharides and proteins (11, 13–15). Studies show that the fucosyltransferase (FUT) enzymes facilitate fucosylation, and dysfunction of these enzymes is linked to metabolic disruptions and various cancers in humans (16–18). Under normal conditions, the gut lumen intermittently produces fucosylated proteins that help human defenses against bacterial infection by blocking the expression of microbial metabolic genes (19–21). The dysregulation of the biological function of the fucosylation process actively contributes to the growth and metastatic invasion of tumors and is a promising target for diagnosing and treating cancer (10–12). A precise mechanism that explains how age-related changes promote core fucosylation in the gut epithelium and subsequently lead to the development of malignant cells in the colon has yet to be established.

Fucosylation mediates several processes in colonic cancer. Patients with metastatic colorectal cancer exhibit elevated levels of tumor markers, sialyl Lewis (sLeX) antigens. The fucosyltransferase 3 (FUT3) gene encodes the Lewis enzyme alpha-1,3/4-fucosyltransferase, which catalyzes the synthesis of sLeX, which is essential for cell adhesion and metastasis (22). Fucosylation with FUT3 and FUT8 alters cellular signaling of the TGF-β receptor (TGFBR). Fucosylation of the TGFBR drives epithelial-mesenchymal transition in CA19-9– and/or sLeX-producing colorectal cancer (CRC) cells (23). Recent studies reveal a “core” fucosylation process with intestinal epithelial cells (IECs) mediated by the FUT8 enzyme. FUT8 transfers GDP-fucose to N-acetylglucosamine of the N-glycan, forming a 1,6-glycosidic bond referred to as core fucosylation (24, 25). IEC core fucosylation regulates the mucosal immune response and host-pathogen interaction (14, 26, 27). A potential strategy that involves minimizing excessive fucosylation might reduce gut pathology and the occurrence of colon cancer in older adults.

Here, we present data from examined colon cancer biopsies obtained from a cohort of patients with colon cancer, both older and younger (see age threshold in Results) individuals, as well as young and old colon cancer mouse models to demonstrate the association between aberrant gut epithelial cell fucosylation and the increasing risk of acquiring colon cancer in older adults. Our data indicate that an imbalance in the symbiotic relationship between the host and gut commensals disrupts fucosylation regulation, influencing the function of epithelial cells and increasing susceptibility to colon cancer in older adults. We evaluated the species-level colonic microbial population to identify pathogenic changes that occur with aging and are responsive to colon cancer. We found that reconstitution of the old fecal microbiota is transmissible to promote colon cancer. Our single-cell RNA-sequencing (scRNA-Seq) results reveal epithelial subsets with considerable fucosylation and a higher colon cancer prevalence in older adults compared with younger. These data suggest the importance of understanding how age-related increases in epithelial fucosylation influence cellular and transcriptome alterations in epithelial-mesenchymal transition to malignant and tumor formation. A better understanding of age-related factors and modulating of the fucosylation process hold promise for new anticancer therapeutics for older patients with colon cancer.

## Results

### Aging is associated with the enhanced fucosylation of IECs.

**Figure 1:**
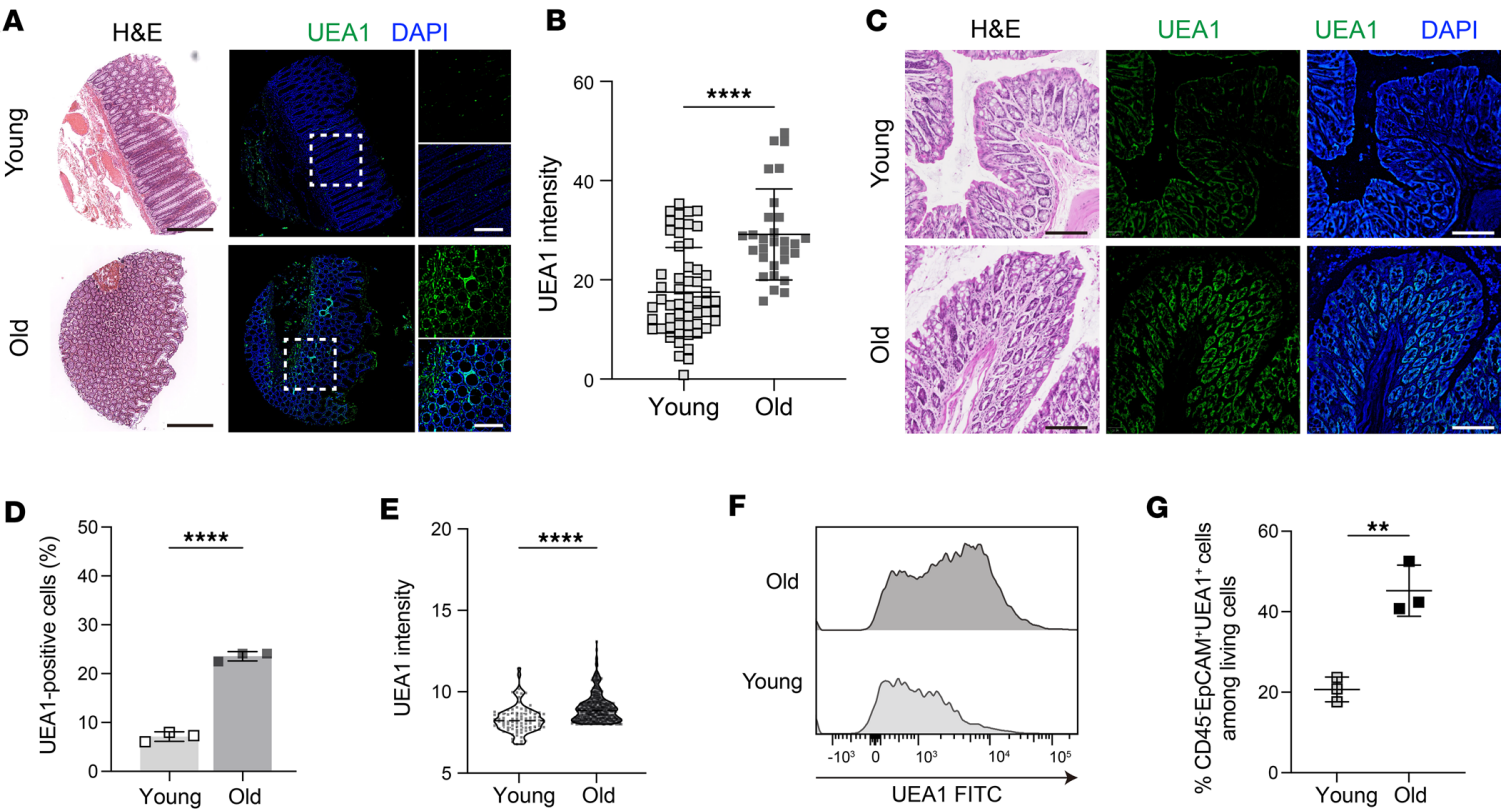
Age-associated changes accelerate the fucosylation process in intestinal epithelium. (**A**) Representative images of hematoxylin and eosin (H&E) staining (left) and immunohistochemical (IHC) staining with UEA1 (right) in colon normal samples (*n* = 85) between old (*n* = 29) and young (*n* = 56) individuals. Scale bar = 500 μm (black) or 200 μm (white). (**B**) The MFI of UEA1 expression across young (<65 years) and old (≥65 years) samples of the cohort shown in **A**. (**C**) Representative images of H&E staining (left) and IHC staining with UEA1 (right) in 8-week (young) and 2-year-old (old) C57BL/6 mouse colons (*n* = 6). Scale bar = 100 μm. (**D** and **E**) The quantification of UEA1^+^ cells (**D**) and the MFI of UEA1 expression (**E**) across mouse colon samples shown in panel **C**. (**F** and **G**) Representative histograms (**F**) and quantification (**G**) of live CD45^–^Epcam^+^ gated UEA1^+^ cells from 8-week-old (young) and 2-year-old (old) C57BL/6 mouse colons detected by flow cytometry. All data represent means ± SD. Unpaired *t* test: **, *P* < 0.01; ****, *P* < 0.0001.

To examine the potential influence of aging on the fucosylation of gut epithelial cells, we first assessed the fucosylation levels in the intestinal epithelium of young and old human and animal models (21, 28). We categorized human participants into 2 groups, young and old, using a predetermined age threshold of 65 years and above for older human adults and below 65 years for young individuals, as established by published research and guidelines from the World Health Organization (29, 30), which included a total of 85 normal samples (29 samples from older adults and 56 samples from young individuals) (Supplemental Figure 1A and Supplemental Table 1; supplemental material available online with this article; https://doi.org/10.1172/jci.insight.167676DS1). We first analyzed the level of fucosylation in normal, noncancerous colons in humans and mice by using ulex europaeus agglutinin-1 (UEA1) staining, a lectin that binds to (1,2)-fucose, to assess the degree of fucosylation in gut epithelial tissue sections (13, 31). The region of interest was delineated through the observation of a fucosylation-positive area and the quantification of UEA1 staining intensity, as shown in the schematics (Supplemental Figure 1, A and B). We observed a significantly positive correlation between age and UEA1 expression (MFI) in normal colon tissue biopsies (Figure 1, A and B, and Supplemental Figure 1C). The sex did not influence the outcome of UEA1 expression, suggesting that age, not sex, is a determinant of epithelial fucosylation under normal conditions (Supplemental Figure 1D). Consistent with findings from humans, we found higher levels of UEA1 expression in the colons of 2-year-old older mice compared with 8-week-old younger mice (Figure 1, C–E). Since UEA1 is a pan-fucosylation stain, we then investigated the levels of UEA1 expression in colonic epithelial (CD45^–^Epcam^+^) cells by flow cytometry (Supplemental Figure 2, A and B). Compared with 8-week-old younger mice, we observed a significantly higher expression of UEA1 in colonic epithelial cells of 2-year-old mice (Figure 1, F and G). These findings suggest that aging is a crucial regulator of fucosylation in gut epithelial cells.

### Increased epithelial fucosylation is linked with a higher risk of human colon cancer in older adults.

**Figure 2:**
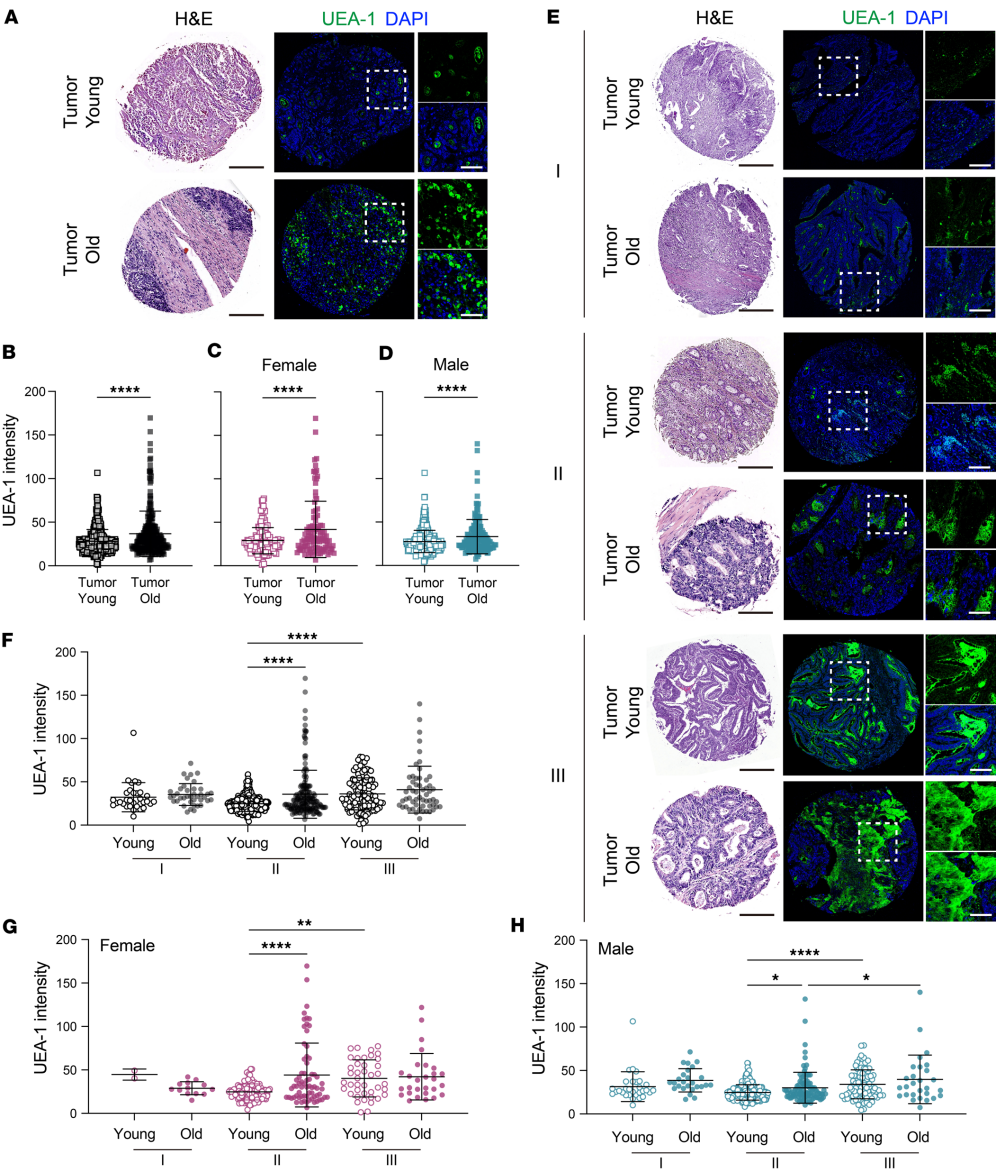
Increased fucosylation is linked with a high risk of colon cancer in older adults. (**A**) Representative images of H&E staining (left) and IHC staining with UEA1 (right) in colon cancer samples between young and older individuals. (**B**–**D**) The MFI of UEA1 expression across young and older samples (**B**) within the cohort depicted in **A** and further differentiated between women (**C**) and men (**D**). (**E**) Representative images of H&E staining (left) and UEA1 staining (right) in colon cancer samples across different cancer stages. (**F**–**H**) The MFI of UEA1 expression across young and older samples at different stages (**F**) within the cohort depicted in panel **E** and further differentiated between women (**G**) and men (**H**). Scale bar = 500 μm (black) or 200 μm (white). All data represent means ± SD. Unpaired *t* test (**B**–**D**) and 2-way ANOVA with Holm-Šídák multiple-comparison test (**F**–**H**): *, *P* < 0.05; **, *P* < 0.01; ****, *P* < 0.0001.

To investigate if aging and fucosylation play a role in the manifestation of colon cancer pathology, we conducted an analysis of fucosylation from the colon cancer human biopsies of young and older adults. A total of 857 human colon biopsies, which were obtained from 528 participants, consisted of 772 cancer samples (267 samples from older adults and 505 samples from young individuals). The samples were collected at different stages of colon cancer, with 9.2% at stage I (*n* = 71), 67.1% at stage II (*n* = 518), and 23.7% at stage III (*n* = 183) (Supplemental Figure 1A and Supplemental Table 1). Our findings revealed the occurrence of dysplasia, hypertrophy, and epithelial damage, as well as UEA1-positive staining, in tumor sections obtained from both young and older adults (Figure 2, A and B). Cumulatively, our data revealed a significant increase in UEA1 expression in tumor sections obtained from older adults compared with young individuals, regardless of sex (Figure 2, B–D). Next, we compared fucosylation levels at various stages of adenocarcinoma progression (stage I, stage II, and stage III) between young and older adults. Interestingly, although fucosylation levels were elevated in both young and older adults during the advancing stages of colon cancer, the increase in fucosylation was quite profound in older adults (Figure 2E). Further analysis showed a significant and extensive increase in fucosylation levels among older individuals with stage II colon carcinoma tissue, compared with levels in young colon cancer biopsies (Figure 2, F–H).

We did not observe sex-dependent changes in UEA1 expression, indicating that age, rather than sex, determines epithelial fucosylation in normal conditions (Figure 2, C, D, G, and H). These findings indicate a potential connection between increased amounts of epithelial fucosylation and the risk of occurrence of colon cancer in older adults. Further research is needed to understand how epithelial fucosylation mechanistically relates to the pathological changes in epithelial cells that lead to colon cancer.

### The cellular and transcriptional changes in the intestinal epithelium with increased fucosylation and cancer-related markers in aging colon cancer.

**Figure 3:**
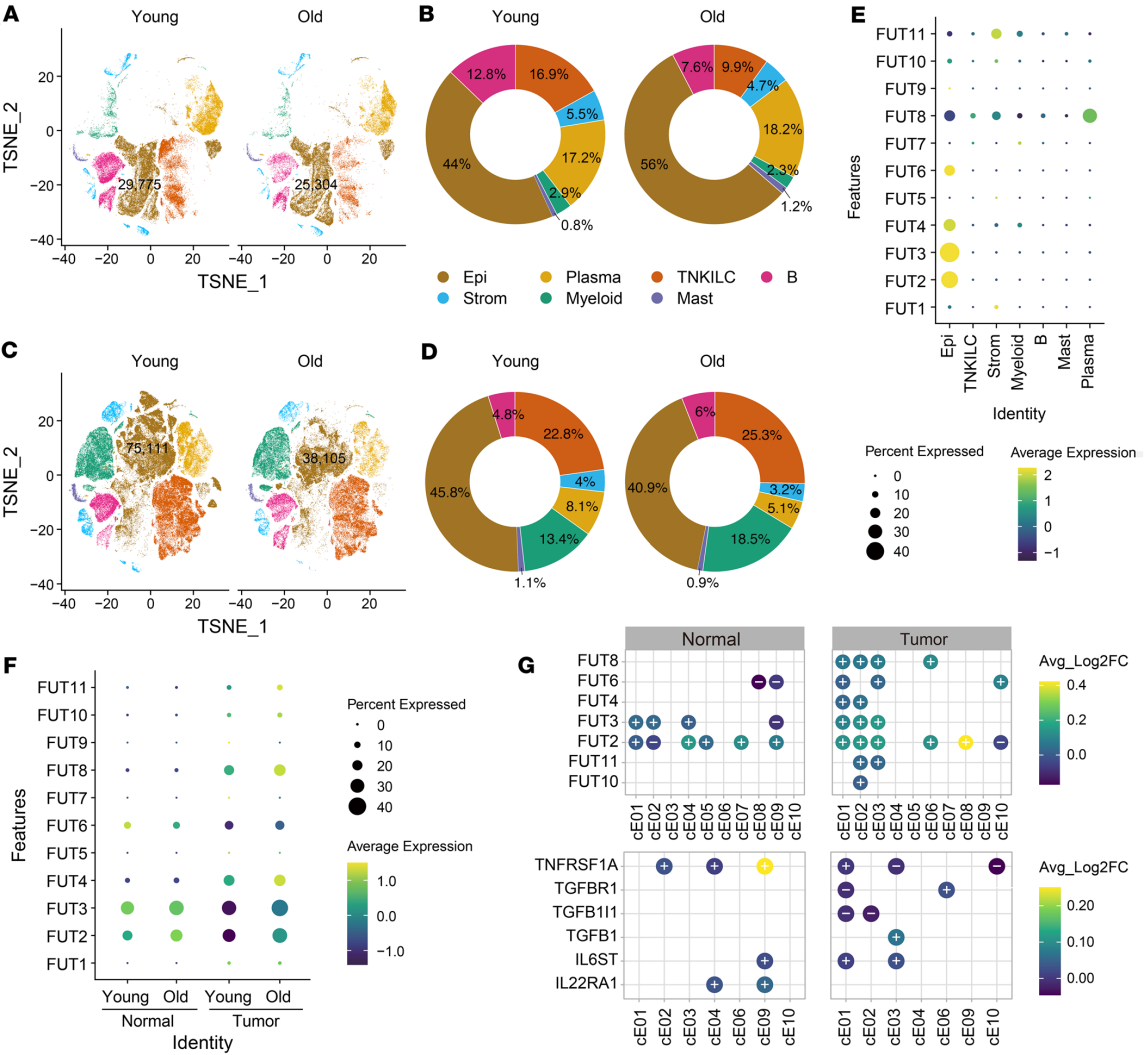
Individuals with colon cancer exhibit higher levels of fucosyltransferase (FUT2, FUT8) gene expression in their colonic epithelial cells. (**A** and **B**) t-Distributed stochastic neighbor embedding (t-SNE) plot of cells from normal patients (**A**) color-coded by cell type as indicated (**B**). Donut chart shows the cell proportion of each cell type among young and old (**B**). TNKILC, T cells, natural killer cells, innate lymphoid cells. (**C** and **D**) t-SNE plot of cells from tumor (**C**) color-coded by cell type as indicated (**D**). Donut chart shows the cell proportion of each cell type among young and old (**D**). (**E**) Dot plot shows the expression pattern of fucosylation genes among all cell types. (**F**) Dot plot shows the expression pattern of the fucosylation genes between young and old across normal and tumor patients. (**G**) Dot plot shows the expression of the significantly differential fucosylation (*FUT2*, *FUT3*, *FUT8*) genes (top) and epithelial repair genes such as TGFB1 and TGFBR1 (bottom) (adjusted *P* < 0.05 and absolute log_2_fold-change > 0.25) between young and old among normal and tumor. A positive symbol (+) indicates increased gene expression, and a negative sign (–) indicates decreased gene expression.

Next, we investigated the heterogeneity of gut epithelial cells to better understand of the specific role of fucosylation in the epithelium and its impact on cellular heterogeneity in individuals with aging and colon cancer (Supplemental Figure 3A). We collected a total of 370,115 cells from colorectal tumors and the adjacent normal tissues of 28 patients with mismatch repair–proficient (MMRp) and 34 patients with mismatch repair–deficient (MMRd) conditions. These cells were obtained from publicly available single-cell data (32). The patients were then divided into 2 groups based on age: the older group (age ≥ 65: 39 patients) and the young group (age < 65: 59 patients) (Figure 3, A and C, and Supplemental Figure 3A). Epithelial tumors display cellular disintegration and reduced cell adhesion (33, 34). A clear reduction was observed in epithelial cell numbers of colon tumor samples from older adults in comparison with younger patients. However, the healthy group did not show this reduction (Figure 3, A–D). We found the epithelial cells were the primary source of the fucosylation genes FUT2, FUT3, FUT4, and FUT6 expression (Figure 3E). The expression of FUT2 and FUT3 in the normal and malignant colon was detected in both young and older adults. Our study revealed a noteworthy increase in FUT8 expression in colon tumors, particularly among older adults (Figure 3F). Furthermore, we conducted a comparative analysis of the expression level in the 2 cancer types among patients of different age groups. We found that FUT2, FUT3, and FUT8 exhibited significantly elevated expression levels in older patients with both cancers when compared with young patients (Supplemental Figure 3B). Next, we assessed the levels of expression in normal colon and tumor tissues for 9 distinct subsets of epithelial cells (cE01–cE09) between young and older adults. We observed a significant increase in the expression of FUT2 in the cE01, cE04, cE05, cE07, and cE09 subsets in normal colon samples and cE01, cE02, cE03, cE06, and cE08 subsets in tumor colon samples. Interestingly, the expression of FUT8 exhibited a significant increase in colon tumor samples cE01, cE02, cE03, and cE06 as a result of the aging process (Figure 3G). Furthermore, we compared the levels of expression of all subgroups of epithelial cells in patients who were MMRp and MMRd, both in young and old patients (Supplemental Figure 3C). The results indicate that the majority of FUT genes, such as FUT1, FUT2, FUT3, FUT4, FUT6, and FUT8, exhibited significant upregulation in older adults.

In addition, we found that expression of interleukin 22 receptor subunit alpha 1 (IL22RA1) was linked with FUT2 expression in the normal older conditions. Compared with young patients with a tumor, we found that the epithelial subset cE03 showed elevated FUT2 and FUT3 and coexpression with tumor-promoting TGFB1 and interleukin 6 cytokine family signal transducer (IL6ST) gene expression in older patients with a tumor. Similarly, we found that increased FUT2 and FUT8 expression was linked to increased TGFBR1 expression in cE06 epithelial subsets (Figure 3G and Supplemental Figure 3D). IL6ST, IL22RA1, TGFB1, and TGFBR1 genes are known to be involved in cellular repair, oncogenesis, and the aging process (35–37). These findings imply that studying the molecular subtypes of scRNA-Seq data could assist us in understanding gene regulatory networks and identifying regulators of colon cancer. Targeted modification of colonic epithelial subset cells associated with high fucosylation and cancer marker expression could be used as a therapy for colon cancer.

### Microbial diversity influences age-related variations in epithelial fucosylation.

**Figure 4:**
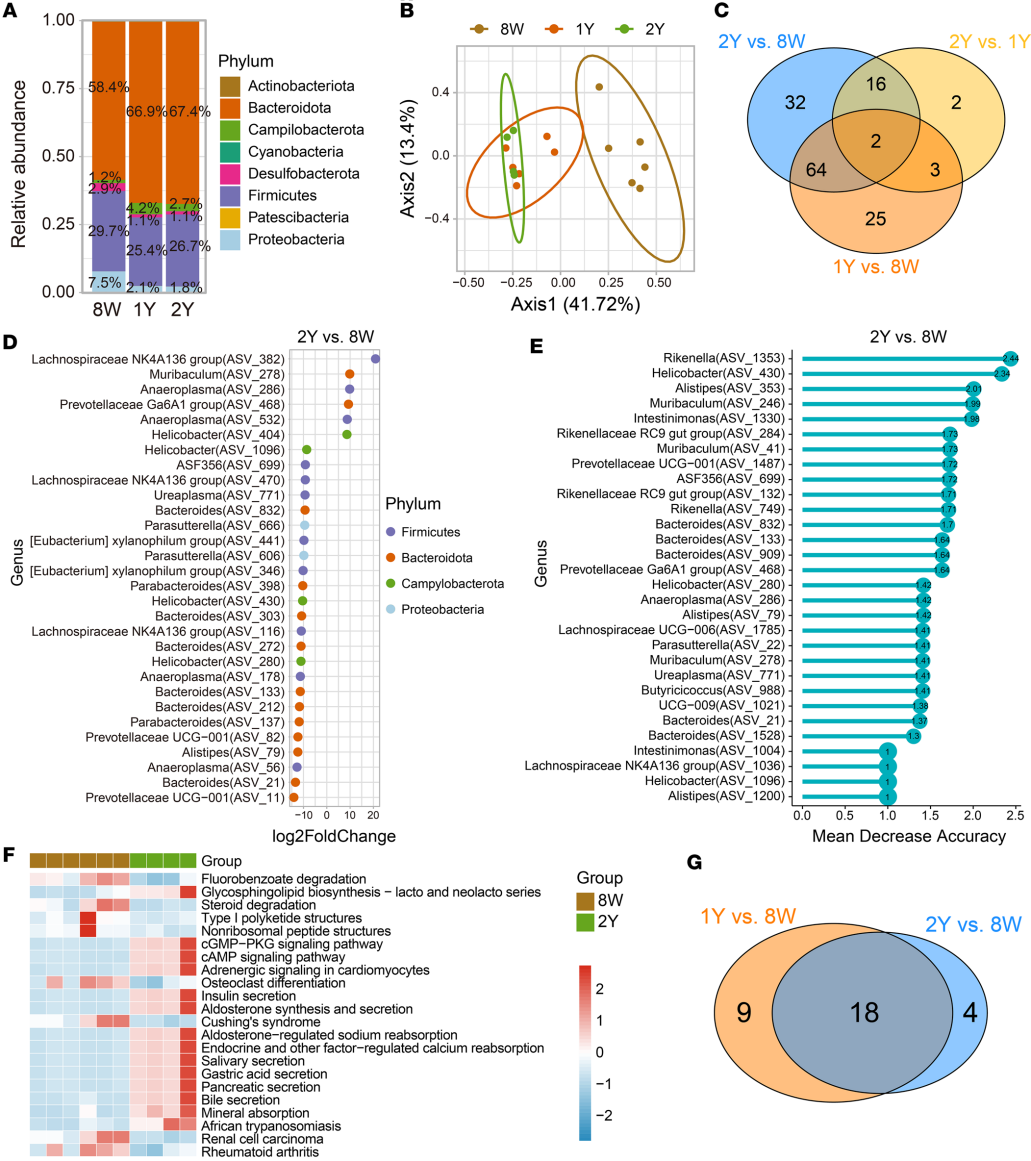
The microbial diversity with aging influences age-related epithelium fucosylation. (**A**) Relative abundance of the most prevalent bacterial phyla among groups. Color stands for phylum level. (**B**) Principal coordinates analysis (PCoA) of the β-diversity based on the Bray-Curtis metric. Colors stand for different groups. (**C**) Venn diagram shows unique or shared significant bacteria from 3 comparisons (1Y versus 8W, 2Y versus 8W, and 2Y versus 1Y). (**D**) Comparison of the significantly differential microbiome at the genus level. Only bacteria with significant differences (adjusted *P* < 0.05 & |log_2_fold-change| > 1) between the 2Y versus 8W mice are shown. Colors stand for phylum level. (**E**) The top 30 differential bacteria distinguish 2Y from 8W mice based on the random forest (RF) model. The bar lengths represent mean decrease in accuracy, indicating the importance of classification. (**F**) Representative heatmap of significant KEGG pathways associated with relative bacterial abundance in 2Y versus 8W mice. The values are scaled by rows (*n* = 6 per group). Only the pathways with significant differences (*P* < 0.05) are shown. (**G**) Venn diagram shows unique or shared significant pathways from 3 comparisons (2Y versus 8W, 2Y versus 1Y, and 1Y versus 8W).

Emerging studies have indicated that gut microbiota plays a crucial role in the regulation of epithelial fucosylation. However, there is evidence linking changes in the diversity of the intestinal microbiota to age-related diseases, metabolic disorders, inflammatory bowel diseases, and colon cancer (13, 26, 38). It is not clear whether age-related microbial alterations are associated with dysregulation of epithelial fucosylation. We conducted a comprehensive analysis of the changes in colonic microbiota in young and older mice. To determine the species-level changes in colonic microbiota in old mice (39), we performed the 16S analysis of fecal material in mice at different age groups, such as 8-week-old (8W: young; *n* = 8), 1-year-old (1Y: middle age; *n* = 8), and 2-year-old (2Y: old, *n* = 8) mice. Compared with 8W young mice (Bacteroidota 58.4%), we found that the abundance of Bacteroidota increased in 1Y (66.95%) and 2Y old mice (67.38%). In contrast, the abundance of Firmicutes decreased in 1Y (25.38%) and 2Y old mice (26.68%) in comparison with 8W young mice (29.7%) (Figure 4A and Supplemental Figure 4A). Prior studies have shown alterations in the relative abundance of Firmicutes and Bacteroidota (F/B) with age (40, 41). However, no difference was found between 1Y versus 2Y old mice. Then, α-diversity analysis was performed to determine the distribution of species abundances among the groups. We observed a higher abundance of microbial diversity in aged (1Y or 2Y) mice with a lower ratio of F/B (1Y: 0.38, 2Y: 0.40) than young mice (0.51) (Figure 4A and Supplemental Figure 4B). Further, principal coordinates analysis (PCoA) showed that 8W mice had clear separation from 1Y and 2Y mice (principal component 1: 41.72%; Figure 4B), which validated the difference among young, middle-aged, and old mice.

Furthermore, we compared the microbiota abundance among all 3 groups and identified the significantly differential bacteria among groups (Figure 4C and Supplemental Figure 4, C and D). We found most of the *Bacteroides* were significantly decreased in 2Y mice, and the *Lachnospiraceae*, *Muribaculum*, *Anaeroplasma*, and *Prevotellaceae* genera increased dramatically in 2Y mice (Figure 4D). In addition, the machine-learning RF analysis was performed to identify the biomarker that significantly contributed to the separation between 2Y and 8W mice (Figure 4E). The *Bacteroides*, such as the bacteria (amplicon sequence variants [ASVs] ASV_21, ASV_133, and ASV_832), were identified as essential biomarkers to determine the difference between 2Y and 8W mice. Notably, the *Bacteroides* were also significantly decreased in 1Y compared with 8W (Supplemental Figure 4C) and identified as biomarkers between 1Y and 8W mice (Supplemental Figure 4E). Moreover, we compared all significant bacteria among all 3 comparisons and found the majority of the significantly differential bacteria (66 ASVs) identified between 2Y versus 8W were shared with 1Y versus 8W mice (Figure 4E). Only 23 bacteria significantly differed between 2Y and 1Y, and only a few biomarkers were identified by RF analysis (Supplemental Figure 4F).

To infer functional biological pathways associated with the disruption in microbial communities, we performed the Kyoto Encyclopedia of Genes and Genomes (KEGG) pathway analysis. We found that the cyclic adenosine monophosphate, cyclic guanosine monophosphate, and insulin pathways become more active in aged mice (Figure 4F and Supplemental Figure 5A). Next, we determined the shared and unique significant pathways among 3 groups (2Y vs. 8W, 1Y vs. 8W, and 2Y vs. 1Y) (Figure 4G). A total of 18 significant pathways were identified from 2Y versus 8W and 1Y versus 8W. No significant pathway was identified between 2Y and 1Y (Figure 4G). Together, our data show that aging-induced changes regulate the balance of gut microbial composition. These changes may be associated with dysregulated fucosylation and metabolic pathways that interact to increase the colon cancer risk in older adults.

### Transplantation of aging microbiota promotes colon cancer pathology in young mice.

**Figure 5:**
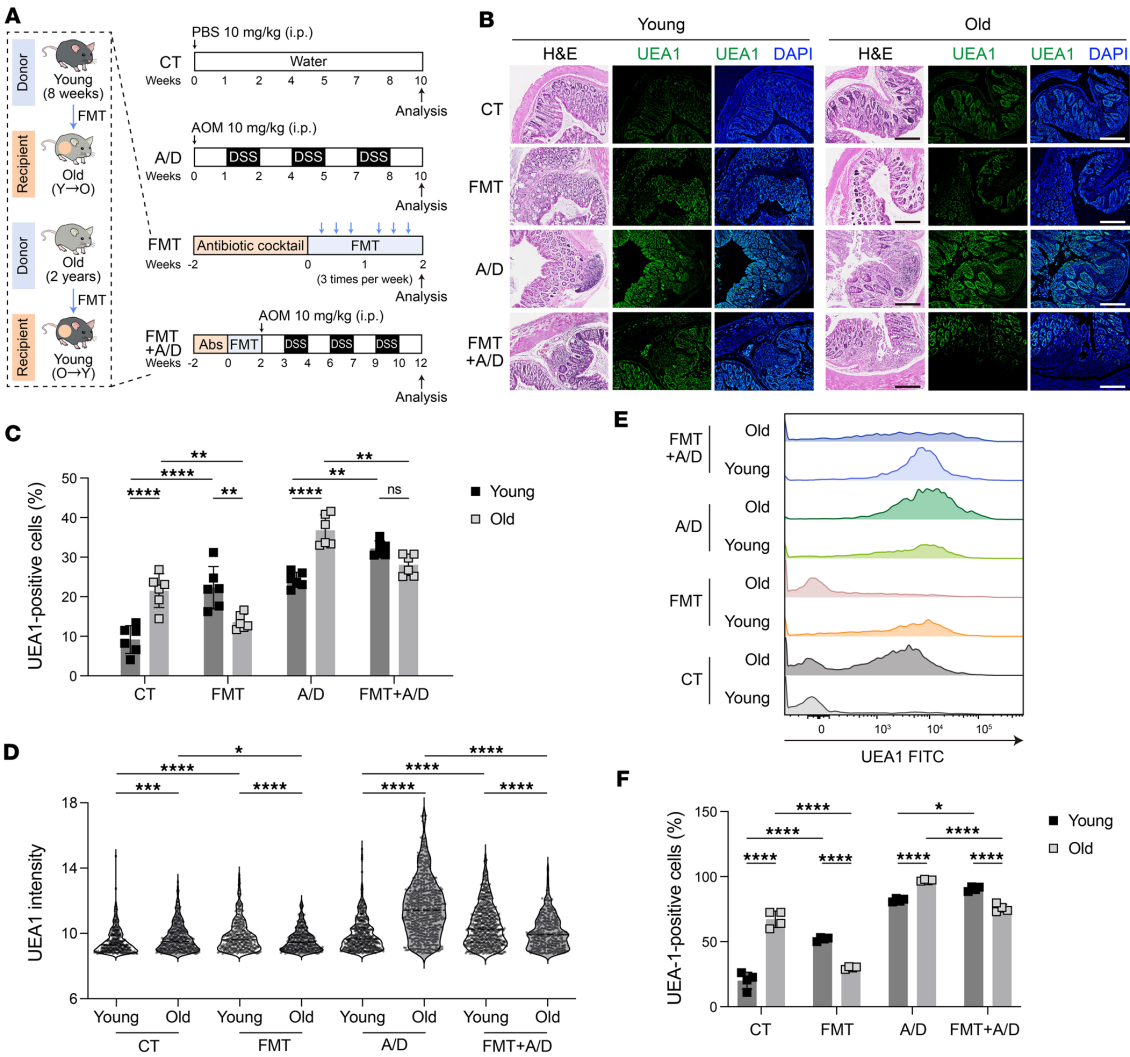
The FMT exhibits a transmissible effect that actively modulates colon cancer pathology in mice. (**A**) Experimental design for FMT to pseudo-germ-free mice and induction of colitis-induced colon cancer using AOM/DSS (A/D) (*n* = 8). Abs, antibiotics. (**B**) Representative images of H&E staining (left) and UEA1 staining (right) of young and old mouse colon samples across control (CT), FMT (Y→O, O→Y), A/D, and FMT (Y→O, O→Y) + A/D groups. Scale bar = 500 μm. (**C** and **D**) The quantification of UEA1-positive cells (**C**) and the MFI of UEA1 expression (**D**) across mouse colon samples shown in **A**. (**E** and **F**) Representative histograms (**E**) and quantification (**F**) of live CD45^–^Epcam^+^ gated UEA1^+^ cells of young and old mouse colon samples across CT, FMT (Y→O, O→Y), A/D, and FMT (Y→O, O→Y) + A/D groups using flow cytometry. All data represent means ± SD. Two-way ANOVA with Holm-Šídák multiple-comparison test: *, *P* < 0.05; **, *P* < 0.01; ***, *P* < 0.001; ****, *P* < 0.0001.

Next, we evaluated the association of aging microbiota with epithelial fucosylation and tumor pathogenesis. To achieve that, we depleted gut microbiota in young and old mice by oral gavage of a broad-spectrum antibiotic cocktail. We then performed fecal microbiota transplantation (FMT) of young mice with the fecal material of older mice and vice versa. Subsequently, control (not antibiotic treated) and fecal transplanted young and old mice were treated with azoxymethane and dextran sodium sulfate (AOM/DSS) to establish colon cancer (Figure 5A). Compared with control young mice, the fecal transplant from old to young (O→Y) mice resulted in higher tumor burden and hypertrophic colon pathology, correlating with increased UEA1-positive cells and UEA1 densities (Figure 5, B–D). In contrast, FMT from young to older recipient mice (Y→O) resulted in ameliorated tumor pathology, which correlated with reduced fucosylation (immunofluorescence staining of UEA1 expression; Figure 5, B–D). To investigate whether the epithelial cells were the target of fucosylation, we detected epithelial cell–specific (CD45^–^Epcam^+^) expression of UEA1 by flow cytometry (Figure 5, E and F). The epithelial specific fucosylation in young (O→Y) and old mice (Y→O) FMT correlated with colon cancer and pathologies, supporting total fucosylation data from immunofluorescence staining of colon sections (Figure 5, B–F). Collectively, these data demonstrate that gut microbiota strongly influences fucosylation and colon tumorigenesis.

### Aging gut microbiota is critical to regulate gut epithelial fucosylation and colon cancer pathology.

**Figure 6:**
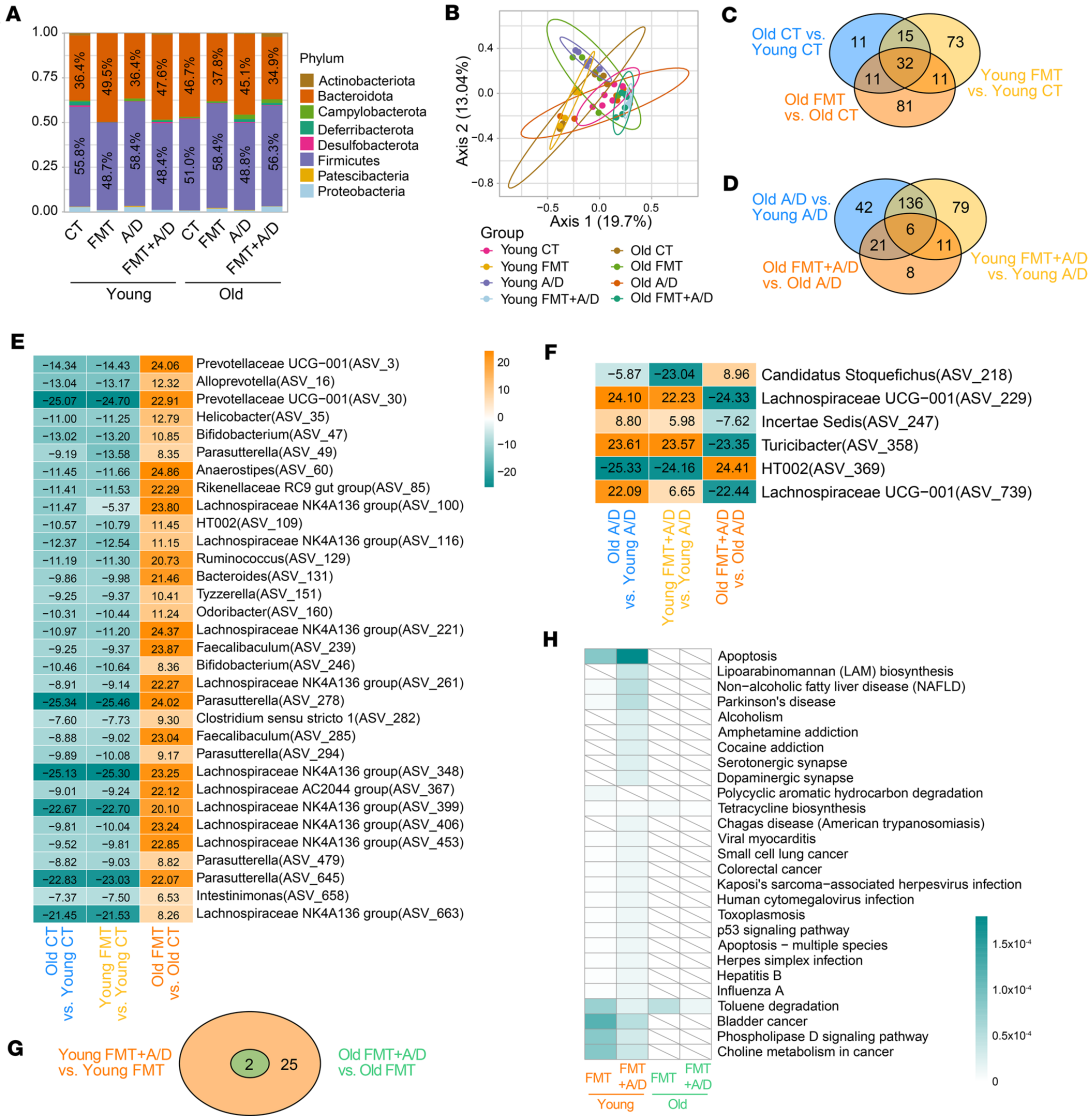
Aging gut microbiota regulates gut epithelial fucosylation and colon cancer pathology. (**A**) Relative abundance of the most common bacterial phyla between populations. The color signifies the phylum. (**B**) PCoA of β-diversity using the Bray-Curtis metric. Colors represent distinct groupings. (**C** and **D**) Venn diagram shows unique or shared significant bacteria from 3 comparisons under normal and AOM/DSS treatments (old versus young, young FMT versus young, and old FMT versus old). (**E** and **F**) Representative heatmap of significant shared microbiome in **C** and **D** at the genus level. Only microorganisms with significant differences (adjusted *P* < 0.05 and |log_2_fold-change| > 1) were shown. Colors represent log_2_fold-change between comparisons. (**G**) Venn diagram shows unique or shared significant pathways from 2 comparisons (young FMT+A/D versus young FMT, old FMT+A/D versus old FMT). (**H**) Representative heatmap of significant shared pathways in **G**. Only pathways with significant differences (*P* < 0.05) were shown. Colors represent log_2_fold-change between comparisons.

To establish the association of microbiota with colon cancer pathologies in young and old mice, we performed 16S microbiome sequencing on fecal samples from AOM/DSS-treated young and old mice (control) as well as FMT young (O→Y) and old (Y→O) mice (Figure 5A). According to the microbiota composition, the FMT young mice (O→Y) had an increased level of Bacteroidota phylum, and a reduced level in Firmicutes phylum,when compared with the young control mice (Figure 6A). Interestingly, we found the FMT old mice (Y→O) showed an increased level of Firmicutes and a decreased level in Bacteroidota phylum when compared with the old control mice (Figure 6A). However, we did not find a significant difference in types of microbiota in both FMT young mice (O→Y) versus young control and FMT old mice (Y→O) versus old control in α-diversity (Supplemental Figure 6). Besides, no difference in α-diversity was observed between young FMT and old control mice, suggesting that FMT young mice mimicked the microbial composition of old mice (Supplemental Figure 6). Similarly, FMT old mice (O→Y) had similar α-diversity to the young control group (Supplemental Figure 6A). Further, PCoA showed the distinct differences of groups in terms of their overall distribution in AOM/DSS-treated control (young and old) as well as FMT young (O→Y) and old (Y→O) mice (Figure 6B).

Further characterization of microbiota identified a total of 32 bacteria that were different between AOM/DSS-treated control (young and old) as well as FMT young (O→Y) and old (Y→O) mice (Figure 6C). The *Bacteroides*, *Prevotellaceae* *UCG-001*, and *Lachnospiraceae* *NK4A136 group*, which showed a dramatic decrease in old mice and reversed with young fecal transplant, showed clear upregulation in old versus young mice and young FMT versus young mice but downregulation in old FMT versus old mice (Figure 6E). Further analysis showed that AOM/DSS-treated groups, old versus young, young FMT versus young, and old FMT versus old, shared 6 significantly differential microorganisms (Figure 6D). These 6 bacterial types were oriented consistently in old versus young, and young FMT versus young, and completely in the opposite manner in old FMT versus old (Figure 6F). Next, we determined the significantly changing pathways between FMT+A/D versus FMT groups within young and old mice (Figure 6, G and H). Interestingly, while 27 significant pathways were enriched between FMT+A/D versus FMT in young mice, such as the p53, Parkinson’s disease, and apoptosis pathways, only 2 significant pathways were identified between FMT+A/D versus FMT within old mice. In contrast, FMT+A/D mice had levels of these pathways indicating they were highly activated (Figure 6H). Together, these data suggest that aging gut microbiota is a crucial regulator of host resistance to colon cancer and associated pathologies via modulating gut epithelial fucosylation.

### Inhibition of fucosylation suppresses the expression of cancer-related genes and metastatic phenotype of colon cancer cells.

**Figure 7:**
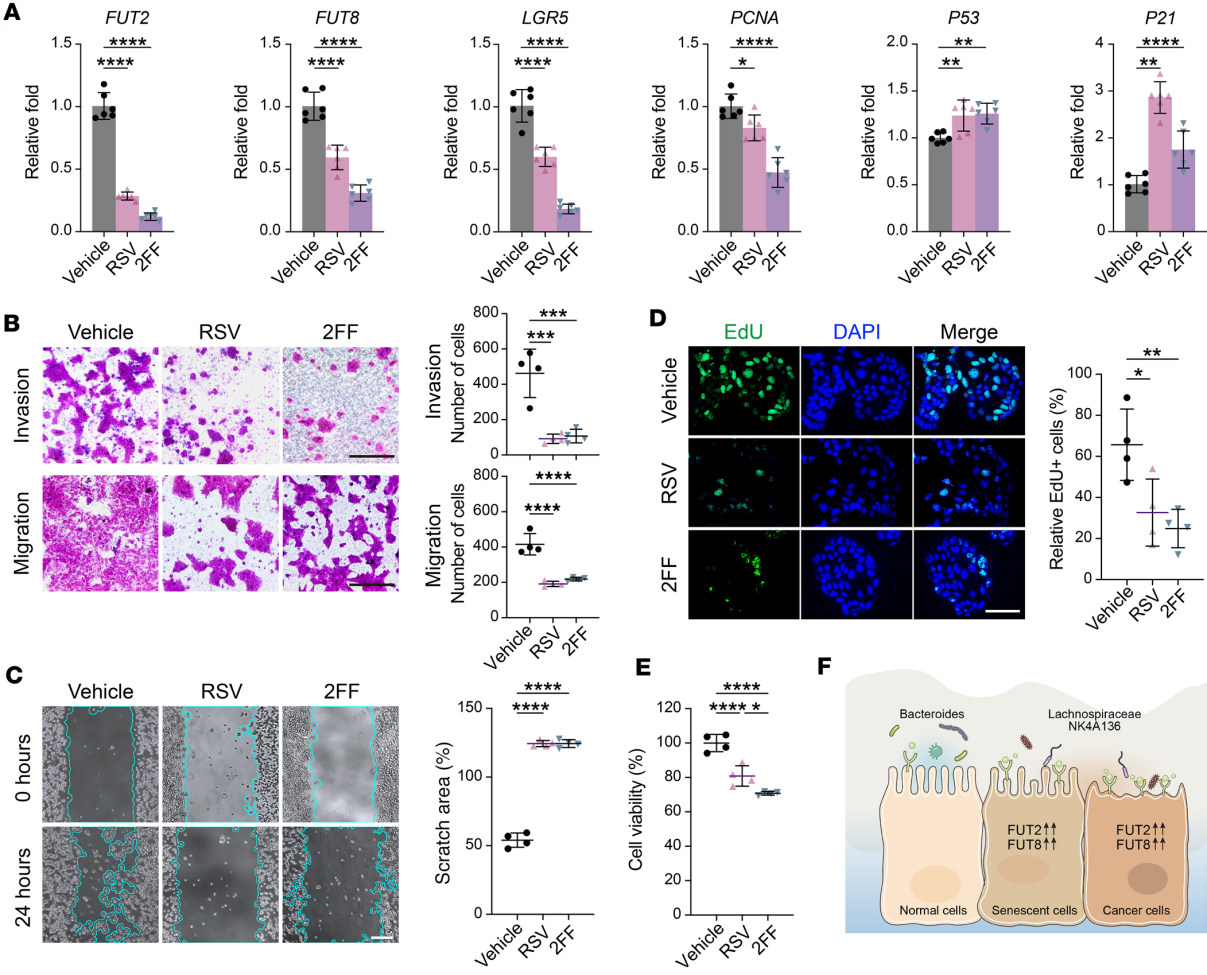
Inhibition of fucosylation suppresses the expression of cancer-related genes and inhibits colon cancer cell migration and proliferation. Human epithelial colon cancer DLD-1 cells were treated with DMSO (vehicle), 100 μM resveratrol (RSV), or 300 μM fucosylation inhibitor 2F-peracetyl-fucose (2FF) for 24 hours. (**A**) Reverse transcription quantitative PCR (qPCR) analysis for *FUT2*, *FUT8*, *LGR5*, *PCNA*, *P53*, and *P21* in DLD-1 cells treated with RSV and 2FF. LGR5, leucine-rich repeat-containing G protein–coupled receptor 5; PCNA, proliferating cell nuclear antigen. (**B**–**E**) DLD-1 cell migration and proliferation inhibited by RSV and 2FF. (**B**) Typical images (left) and corresponding statistical results (right) of Transwell invasion and migration assays in DLD-1 cells treated with vehicle, RSV, and 2FF. Scale bar = 50 μm. (**C**) Representative images (left) and quantification (right) of wound healing assay at 0 and 24 hours in DLD-1 cells treated with vehicle, RSV, and 2FF. Light blue line marks scratch wound edges. Scale bar = 200 μm. Scratched areas were quantified as a percentage after 24 hours relative to 0 hours. (**D**) Representative images (left) and relative fraction (right) of EdU-positive cells. EdU, 5-ethynyl-2′-deoxyuridine. Scale bar = 50 μm. (**E**) Measurement of cell proliferation using CCK8 assays. CCK8, cell counting kit-8. All data represent means ± SD; *n* = 6 (**A**) and *n* = 4 (**B**–**E**). *, *P* < 0.05; **, *P* < 0.01; ***, *P* < 0.001; ****, *P* < 0.0001 by 1-way ANOVA. (**F**) Schematic of proposed mechanism illustrating how core fucosylation of IECs influences microbiota and may impact aging and cancer development.

To investigate the direct contribution of fucosylation on epithelial metastatic function in vitro, we treated human colon cancer epithelial cells (DLD-1) with a pan-fucosylation inhibitor, 2F-peracetyl-fucose (2FF) (42). The treatment of DLD-1 cells with 2FF reduced the expression of *FUT2*, and *FUT8*, suggesting that 2FF treatment inhibited the epithelial fucosylation (Figure 7A). The inhibition of fucosylation resulted in the downregulation of *LGR5* and *PCNA* and the upregulation of *P53* and *P21* genes associated with the cell cycle and development of metastatic phenotype (Figure 7A). Next, we treated DLD-1 cells with RSV, a plant-derived polyphenol associated with longevity and anticancer properties (43, 44), to investigate its effect on epithelial fucosylation. We found RSV significantly reduced *FUT2* and *FUT8*, which correlated with the downregulation of *LGR5* and *PCNA* and upregulation of *P53* and *P21* genes (Figure 7A). Further, both RSV and 2FF produced similar effects on cell migration, proliferation, and invasion, suggesting that RSV-mediated changes in malignant epithelial cells are mediated via the regulation of fucosylation (Figure 7, B–E). The results validate our hypothesis that RSV has a protective effect against cancer by potentially inhibiting fucosylation in malignant epithelial cells. RSV could potentially be used as a therapeutic agent for advanced age-related colon cancer, specifically by addressing long-term and persistent fucosylation associated with aging. Our model suggests that the dysregulated fucosylation of aging epithelial cells, caused by the imbalance of *FUT2* and *FUT8* expression, increases the risk of colon cancer in older adults. Managing a healthy host-commensal interaction could be an effective strategy to regulate epithelial fucosylation and reduce colon cancer pathology (Figure 7F).

## Discussion

Fucosylation represents a critical posttranslational modification of glycoproteins and glycolipids, actively contributing to gut epithelial cell function and the maintenance of gut homeostasis. The dysregulation of fucosylation is believed to contribute to the worsening of colitis and cancer conditions, although the underlying mechanism is not yet fully understood (10–12). In this study, we establish a causal relationship between age-specific dysregulation of host-commensal symbiotic relationship with elevated fucosylation of IECs and colon cancer. Our in vitro data support the in vivo findings from human and mouse models of colon cancer. Notably, our findings have revealed that the occurrence of gut epithelial fucosylation markedly increases as individuals age, thereby increasing the risk of malignancies in older adults.

Our findings show a statistically significant link between age and sustained epithelium fucosylation in older adults and animals compared with young at steady state. To investigate the potential impact of alterations to gut epithelial fucosylation on cellular dysfunction and the development of CRC in older adults, we conducted an in-depth analysis of colon cancer and control tissue samples obtained from both male and female individuals. Notably, we found that the levels of fucosylation increased in both young and older adults in colon cancer conditions. However, the extent of fucosylation was substantially lower in the young colon cancer conditions compared with older patients with colon cancer. We also noticed that older patients in early stage II of colon cancer had the highest fucosylation levels compared with younger adults. We found a consistent pattern of fucosylation that progressively intensified with age across different stages of colon cancer. These findings suggest that fucosylation levels could serve as a dependable biomarker for monitoring the development of colon cancer. Previous studies have established a strong correlation between the progression of colon cancer to advanced stages and the occurrence of epithelial integrity loss and hyperplasia in the later stage of colon cancer (33, 34). This could be a plausible rationale for the increased although not significant variations in fucosylation patterns during the advanced stages of colon cancer. Further investigations into a larger group of young and old patients with colon cancer across different tumor stages will help address the differences.

Gut epithelial cells exhibit considerable heterogeneity and can be categorized into various subsets, each exhibiting unique functional attributes in colon cancer conditions. We additionally identified complex differences in fucosylation levels in different types of epithelial cells in young and old patients with colon cancer. Our analysis using scRNA-Seq data provides a more detailed understanding of the diversity within the colon epithelium and its relevance to the fucosylation of epithelial subsets in the progression of colon cancer. Therefore, we examined the level of fucosylation in colonic epithelial cells. Our findings reveal specific epithelial subpopulations expressing different degrees of fucosylation, suggesting that certain epithelial subsets might be more dominantly associated with the fucosylation and, consequently, the manifestation of colon cancer. Further characterization identified the pattern of fucosylation in epithelial subpopulations and the presence of cancer-related markers among older and young patients with colon cancer. Intriguingly, the tumor-promoting FUT2 and FUT8 expression was highly expressed in tumor colon cE02, cE03, and cE06 epithelial subsets in aging colon tumor. Increased FUT2 and FUT8 expression has been linked to cancer (45, 46). Additionally, older patients with a tumor exhibited elevated FUT2, FUT3, and FUT8 coexpression with tumor-promoting IL6ST, IL22RA1, TGFB1, and TGFBR1 genes compared with young tumor. TGFB1 produced by senescent cells promotes cell survival and epithelial regeneration (47–49). Our findings, in conjunction with previous studies, show that the signaling pathway involving IL22 and TGFB1 helps establish a microenvironment that promotes epithelial-mesenchymal transitions, malignancy progression, and tumor formation (22, 23, 50). However, further functional studies are required to establish a relationship between dysregulated fucosylation on specific epithelial subsets that are involved in the increased epithelial-mesenchymal transition process and mediated risk for metastasis in older adults. These findings suggest that a potential treatment strategy for older patients with colon cancer could involve targeting the specific subset of colonic epithelial cells that are responsible for increased fucosylation and cancer markers. Considering the presence of a symbiotic association between host microorganisms and the necessity of fucosylation for metabolizing fucosylated glycans as a nutritional resource, it is critical to determine whether microbial dysbiosis is the underlying factor contributing to the observed rise in fucosylation. In turn, this may increase susceptibility to the risk of colon cancer. Subsequently, we aim to examine the potential correlation between the prevalence of host organisms within the intestinal tract and age and its potential impact on the dysregulation of gut fucosylation. Interestingly, in line with previous research (51, 52), we have noted a reduction in the prevalence of *Bacteroides* and a noteworthy rise in the quantities of *Lachnospiraceae*, *Muribaculum*, *Anaeroplasma*, and *Prevotellaceae*. These microbial taxa have been associated with elevated levels and linked to pathological outcomes in older mice (53–55). Subsequently, to infer functional biological processes relevant to cancer-causing aging in microbial communities, we performed a KEGG pathway analysis and uncovered the critical pathways across the 3 comparisons that increase the risk of colon cancer in older adults. Based on the results, it can be inferred that the presence of age-related microbial diversity and metabolic abnormalities in a homeostatic condition considerably contributes to the greater vulnerability of aging animals to colon cancer.

To demonstrate the influence of aging microbial communities on epithelial fucosylation, tumor microenvironment, and tumor pathogenesis, we reconstituted the feces from old mice into young mice (O→Y) pretreated with antibiotics. Our findings indicate that in an AOM/DSS-induced colon cancer model, the O→Y FMT increased tumor burden and colon pathology; conversely Y→O FMT exerted a protective effect against colon pathology and tumor burden. These findings suggest that alterations in age-dependent gut microbial compositions markedly regulate pathological epithelial fucosylation, promoting colon cancer and colonic pathologies.

Following the determination of the impact of aging and microbial dysbiosis on the process of epithelial fucosylation, we next investigated if the blockade of fucosylation could reverse the metastatic phenotype of cancer epithelial cells. Further, we postulated that RSV, which has a proven antiaging effect, would suppress epithelial fucosylation and mitigate the metastatic phenotype of cancer epithelial cells. Notably, the blockade of fucosylation by 2FF or RSV treatment suppressed cell migration, evasion, and proliferation of cancer epithelial cells. These findings suggest that an age-related increase in epithelial fucosylation is associated with an elevated risk of developing colon cancer and that the suppression of fucosylation is a potential therapeutic approach to mitigate age-associated colonic pathologies and cancer.

Overall, it is crucial to establish how the aging process increases the vulnerability to colon cancer and other colonic pathologies. Our findings suggest that the changes in epithelial fucosylation should be considered a potential therapeutic approach against age-associated colonic pathologies and warrant future studies to advance the field.

## Methods

### Sex as a biological variable.

Sex as a biological variable was addressed, and an equal number of male and female mice was used in all experiments. Mice of both sexes were randomly assigned to experimental groups at various time points based on their ages (8 weeks, 1 year, and 2 years).

### Human colon samples.

Human colon normal (85 samples from 85 participants) and cancer (772 samples from 443 participants) samples were obtained from tissue microarray (TMA; CO487, CO1201, CO1504, and CO6161, Biomax). The main characteristics of patients and tumors are listed in Supplemental Tables 1 and 2.

### Laboratory animals.

C57BL/6 female and male mice were purchased from The Jackson Laboratory. All animals were kept and bred in the University of North Dakota (UND) animal facility and treated in accordance with the Institutional Animal Care and Use Committee’s (IACUC) and NIH’s *Guide for the Care and Use of Laboratory Animals* (National Academies Press, 2011) standards. Mouse fecal material was aseptically obtained and processed at the UND Genomics facilities.

### Histology and IHC.

The H&E-stained images of TMA were obtained from Biomax online. Mouse colon samples were extracted, fixed with 10% formalin for 24 hours, and embedded in paraffin. Sections (5 μm) were deparaffinized and stained with H&E. For IHC analysis, the TMA and mouse colon slides were deparaffinized, blocked by 5% fetal bovine serum (FBS) for 30 minutes in the dark at room temperature, and stained with UEA1 Fluorescein (FL-1061, Vector Laboratories) in the dark at 4°C overnight. After incubation, slides were washed with 1× phosphate-buffered saline (PBS) media and applied with a single drop of mounting media (Fluromount-G, with DAPI; 00-4958-02, Thermo Fisher Scientific). All pictures were collected using an Olympus Total Internal Reflection Fluorescence microscope equipped with a Hamamatsu ORCA-Flash4.0 camera at the UND Histology core. The images were analyzed in batches using QuPath software (version 0.4.4) for the visualization, cell segmentation, positively stained (UEA1^+^) cell quantification, and MFI in the identification of the cells, as previously described (56, 57) (Supplemental Table 3).

### Flow cytometry.

Single-cell suspensions were prepared from the mouse colon as described in our published report (50, 58). Briefly, colons were longitudinally opened in ice-cold HBSS (21020-CV, Corning), washed, and digested in a solution containing collagenase type IV (1088866001, Roche) and DNase I (D263–5vl, Roche) at 37°C for 20 minutes with moderate rotation. The digestions were vortexed for 20 seconds after incubation. Cell suspensions were strained by passing through a 40 μm cell strainer (Falcon, Corning). Single-cell suspensions were incubated with stain from a LIVE/DEAD Fixable Blue Dead Cell Stain Kit (L23105, Invitrogen), Epcam-PE (12-9326-42, eBioscience), CD45-PerCP-Cy5.5 (103132, BioLegend), and UEA1-FITC (FL-1061, Vector Laboratories). We acquired 100,000 events per sample using a FACSymphony Flow Cytometer (BD Biosciences) and analyzed them using FlowJo software (version 10.4.0), according to the gating strategy (Supplemental Figure 2A), with fluorescence-minus-one gating or fully stained samples (Supplemental Figure 2B).

### Microbiome analysis.

Mouse fecal genomic DNA was extracted, and 16S rRNA was sequenced as previously described (59). The raw paired-end FASTQ files were processed by the microbial package (https://github.com/guokai8/microbial), which integrated multiple functions from DADA2 (60), phyloseq (61), DEseq2 (62). Data processing was carried out by “processSeq” function, which contained read trimming (Read1 and Read2 were trimmed at 210 and 170 base positions, respectively) and taxonomy classification (based on Silva database version 138). The data were filtered with the “prefilter” function (*min = 10*, *perc = 0.3*).

Relative abundance was calculated based on the percentage at the phylum level. The α-diversity was assessed using “Observed,” “Shannon,” and “Simpson” index. For β-diversity, PCoA plot based on Bray-Curtis dissimilarity was used with proportionally normalized data. Differential abundance analysis was done with adjusted *P* < 0.05 and |log_2_fold-change|>1 to identify significant ASVs. Biomarker selection was implemented using the function “biomarker” from the microbial package. Next, we used Tax4Fun2 package (63) based on Ref99NR database mode and Tax4Fun2_ReferenceData_v2 to predict significant KEGG pathways and functions (*P* < 0.05 and |log_2_fold-change| > 1).

### Differential expression analysis by scRNA-Seq data analysis.

The publicly available scRNA-Seq data and corresponding patient data were collected from the National Center for Biotechnology (NCBI) Gene Expression Omnibus (GEO) database (accession GSE178341) (32). The scRNA-Seq data were loaded into R and normalized with Seurat package. The cell types were defined based on the results from Pelka et al. (32). MAST (64) was used to conduct differential analysis on each cell type to find the genes that were differentially expressed between the young and old groups with the adjusted *P* < 0.05.

### AOM/DSS colon cancer model.

Sex-matched young (8 weeks) and old (2 years) C57BL/6 mice of both male and female sexes were selected for the study (*n* = 8). The mice were i.p. injected with 10 mg/kg AOM (Merck). One week later, mice were fed 3 rounds of DSS (MP Biomedicals) to mimic CRC. For each cycle, mice were allowed free access to water containing 2% DSS for 1 week with 2 weeks of ordinary water for recovery. Mice of control group received an i.p. injection of PBS and were fed with distilled water. All the mice were euthanized after 3 cycles of DSS. Fecal samples were collected immediately for analysis of microbiota, and colon tissues were collected for histological analysis.

### Antibiotic treatment and FMT.

Mice received antibiotics cocktail of 10 mg/mL ampicillin (A1593, MilliporeSigma), 10 mg/mL metronidazole (PHR1052, MilliporeSigma), and 5 mg/mL vancomycin hydrochloride (SBR00001, MilliporeSigma) in drinking water for 2 weeks to deplete the gut microbiota. For ex vivo FMT, feces from donor young (8 weeks) and old (2 years) mice were collected and stored at –80°C. C57BL/6 mice treated with antibiotics were moved to sterile cages that had been autoclaved. The mice received an oral gavage of 300–400 μL of a suspension made by homogenizing fecal pellets from untreated mice in water. Control mice were orally administered either water or homogenates made from their own excrement. To find the role of microbiota in colon cancer, we treated these mice with AOM/DSS to develop colon cancer.

### Cell culture.

DLD-1 cells (human colon cancer cell line, CCL-221, ATCC) were gifted from Min Wu (UND) and cultured in RPMI-1640 medium (A1049101, Gibco) with 10% FBS with 1× penicillin-streptomycin solution (15140163, Gibco) at 37°C in a 5% CO_2_ incubator for a maximum of 10 passes.

### RNA isolation and qPCR.

DLD-1 cells were cultured overnight in 6-well (5 × 10^5^ cells/well) plates and treated with 100 μM antiaging RSV (R0071, TCI America) or 300 μM 2FF (HY-W096600, MedChemExpress) for 24 hours for RNA isolation. The DMSO concentration in all experiments, including vehicle control, was no more than 0.1%. Total RNA was isolated from DLD-1 cells with TRIzol reagent (15596018, Ambion, Thermo Fisher Scientific). cDNA was generated using a High-Capacity cDNA Reverse Transcription Kit (4368814, Thermo Fisher Scientific). qPCR was performed using 2× SYBR Green dye (B21202, Bimake) on the CFX Connect real-time PCR detection system (Bio-Rad). Genes were normalized to *GAPDH* using 2^-ΔΔCT^ method. The primer sequences are as follows: *FUT2* F: 5′-TCCCCTGGCAGAACTACCA-3′; *FUT2* R: 5′-GGTGAAGCGGACGTACTCC-3′; *FUT8* F: 5′-AACTGGTTCAGCGGAGAATAAC-3′; *FUT8* R: 5′-TGAGATTCCAAGATGAGTGTTCG-3′; *LGR5* F: 5′-CTCCCAGGTCTGGTGTGTTG-3′; *LGR5* R: 5′-GAGGTCTAGGTAGGAGGTGAAG-3′; *PCNA* F: 5′-CCTGCTGGGATATTAGCTCCA-3′; *PCNA* R: 5′-CAGCGGTAGGTGTCGAAGC-3′; *P53* F: 5′-CAGCACATGACGGAGGTTGT-3′; *P53* R: 5′-TCATCCAAATACTCCACACGC-3′; *P21* F: 5′-TGTCCGTCAGAACCCATGC-3′; *P21* R: 5′-AAAGTCGAAGTTCCATCGCTC-3′; *GAPDH* F: 5′-GGAGCGAGATCCCTCCAAAAT-3′; and *GAPDH* R: 5′-GGCTGTTGTCATACTTCTCATGG-3′.

### Cell invasion and migration assays.

For invasion assay, a 24-well Transwell chamber (Corning) was precoated with Matrigel (BD Biosciences). DLD-1 cells (4 × 10^5^) were cultured on the upper chamber with 200 μL medium without FBS, and 600 μL medium with 20% FBS was filled in the lower chamber. For migration assay, cells were cultured using the same invasion protocol. After 2 days, migrated cells were stained with 0.1% crystal violet (Ricca) and counted.

### Wound healing assay.

DLD-1 cells were cultured overnight in 6-well (5 × 10^5^ cells/well) plates. Scratches were created by manually scraping the cell monolayer with a 200 μL pipette tip. Cells were treated with DMSO (vehicle), 100 μM RSV, or 300 μM 2FF. The scratch area was imaged at 0 and 24 hours using an EVOS FL digital inverted microscope (Thermo Fisher Scientific) and quantified using ImageJ (Fiji).

### EdU and CCK8 assays to detect cell proliferation.

We used EdU detection kit (C0075S, Beyotime). A total of 50 μM EdU medium was prepared, and 200 μL was added to each well for 2 hours. The wells were then subjected to Apollo staining for 30 minutes at room temperature, following the manufacturer’s instructions, and images were obtained using a fluorescence microscope (original magnification, ×200). The relative proportion of EdU-positive cells was quantified using ImageJ. Cell viability was measured by CCK8 assay kit (C0038, Beyotime). DLD-1 cells were cultured overnight in 96-well (5,000 cells/well) plates and treated with DMSO (vehicle), 100 μM RSV, or 300 μM 2FF for 24 hours. CCK8 reagent was then added to each well. Absorbance was read at 450 nm after 4 hours, and the relative cell viability rate was calculated.

### Statistics.

All data are represented as means ± SD. Unpaired 2-tailed *t* test was used for 2-group comparison, and 1-way ANOVA or 2-way ANOVA was used between multiple groups, performed with GraphPad Prism (version 9.3.1). Statistical analysis was also carried out in R (version 4.0.2). *P* < 0.05 was considered statistically significant.

### Study approval.

All animal experiments were approved by the UND IACUC. Human colon samples were obtained from TMA (CO487, CO1201, CO1504, and CO6161, Biomax). Human scRNA-Seq data were obtained from the NCBI GEO database with accession number GSE178341.

### Data availability.

Values for all data points are available in the Supporting Data Values file and from the corresponding author upon request. The 16S rDNA-Seq raw data have been deposited into the NCBI Sequence Read Archive (BioProject ID: PRJNA887718, PRJNA1049455).

## Author contributions

RM conceived the project and designed experiments. ZW, PG, and KG analyzed and interpreted data. ZW, ALDC, MR, HM, JF, SJ, GS, JSG, JW, and TD performed experiments. RM, ZW, and KG organized the figures and wrote the manuscript. RM, ZW, KG, JH, RS, HMBB, NK, and DAJ revised the manuscript. All authors provided comments on the manuscript.

## Supplementary Material

Supplemental tables 1-3

Supporting data values

Supplemental data

## Version 1 01/30/2024

In-Press Preview

## Version 2 03/08/2024

Electronic publication
