# Supplementary material for: Age-related dysregulation of intestinal epithelium fucosylation is linked to an increased risk of colon cancer
Source: JCI Insight. 2024 Jan 30;9(5):e167676. doi: 10.1172/jci.insight.167676 (PMC10972600; doi:10.1172/jci.insight.167676)
Supplement: Supplemental data [file jciinsight-9-167676-s101.pdf]

Supplementary Materials for

Age-related dysregulation of intestinal epithelium fucosylation is linked to an increased risk of colon cancer

Supplemental Data includes:

Supplemental Figure 1-6

Supplemental Figure Legends

Supplemental Table 1-3

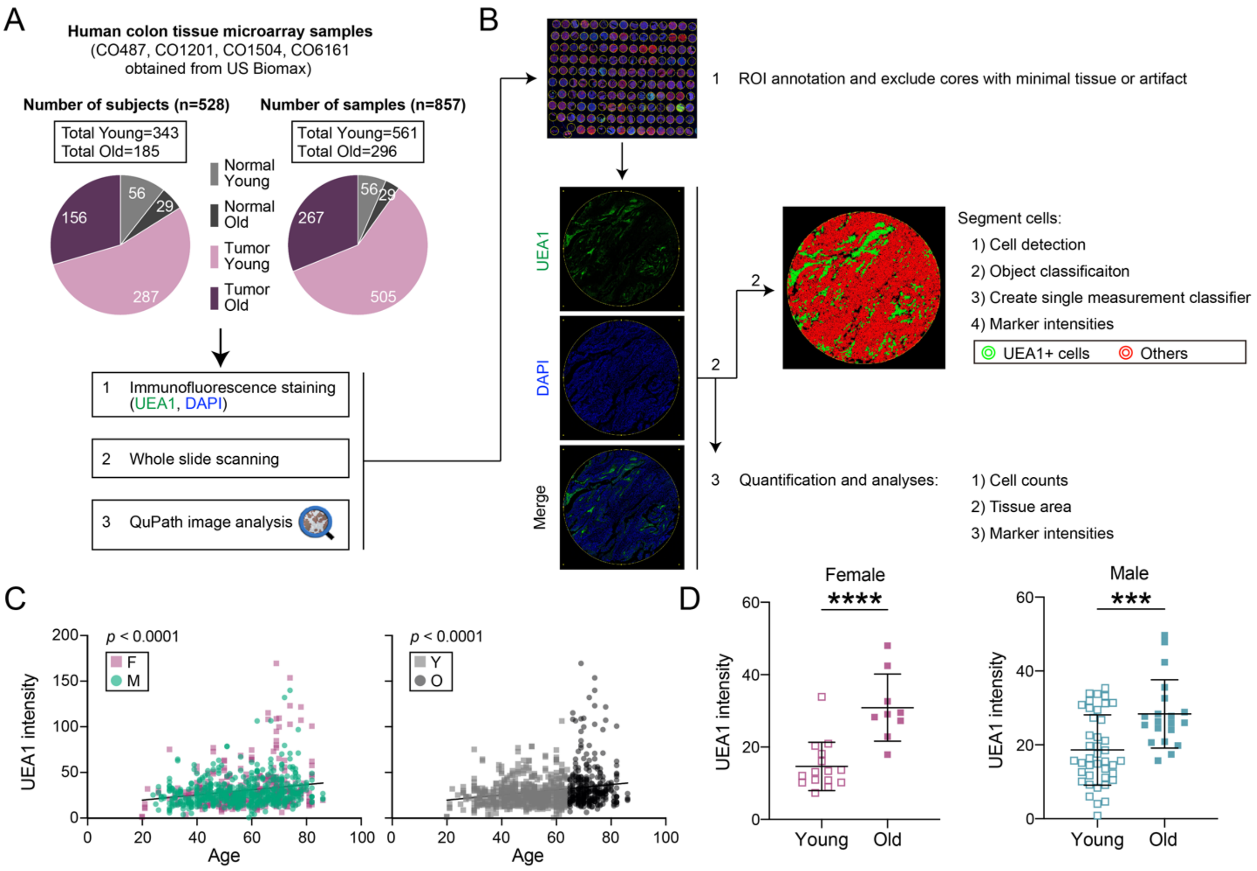

Supplemental Figure 1. Workflow for immunohistochemistry analysis of colon samples. (**A**) Overview of human colon tissue microarray cohort characteristics. 85 normal samples from 85 individuals and 772 cancer samples from 443 individuals were detected in our study. (**B**) Flow diagram highlights the steps used for image optimization and analysis, including staining, scanning, and analyzing (regions of interest selecting, nuclei identification, and cell segmentation performed by QuPath). (**C**) Pearson correlation analysis of UEA1 intensity in colon cohort and host age among sex (left) and age (right). n=294 (females) or 563 (males). (**D**) The mean fluorescence intensity of UEA1 expression across young and old samples differentiated between females (left) and males (right). n=561 (Young) or 296 (Old). All data represent means ± SD; Unpaired t-test: ***p<0.001, ****p<0.0001.

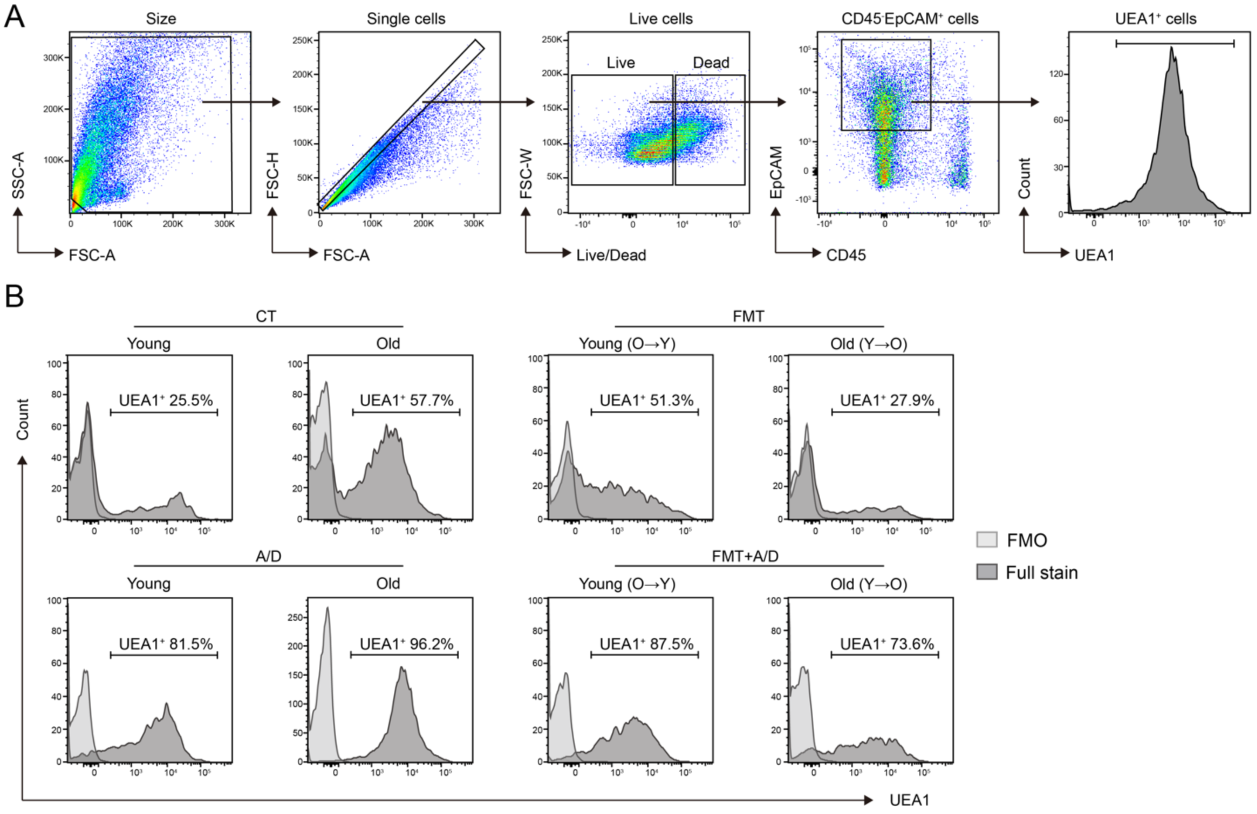

Supplemental Figure 2. UEA1 expressing gut epithelial cell profiles using flow cytometry. (**A**) Gating strategy of live $CD45^{-}Epcam^{+}UEA1^{+}$ cells. (**B**) Fluorescence-minus-one (FMO) controls for UEA1. Graphs are paired with the full stain in dark grey and the FMO in light grey.

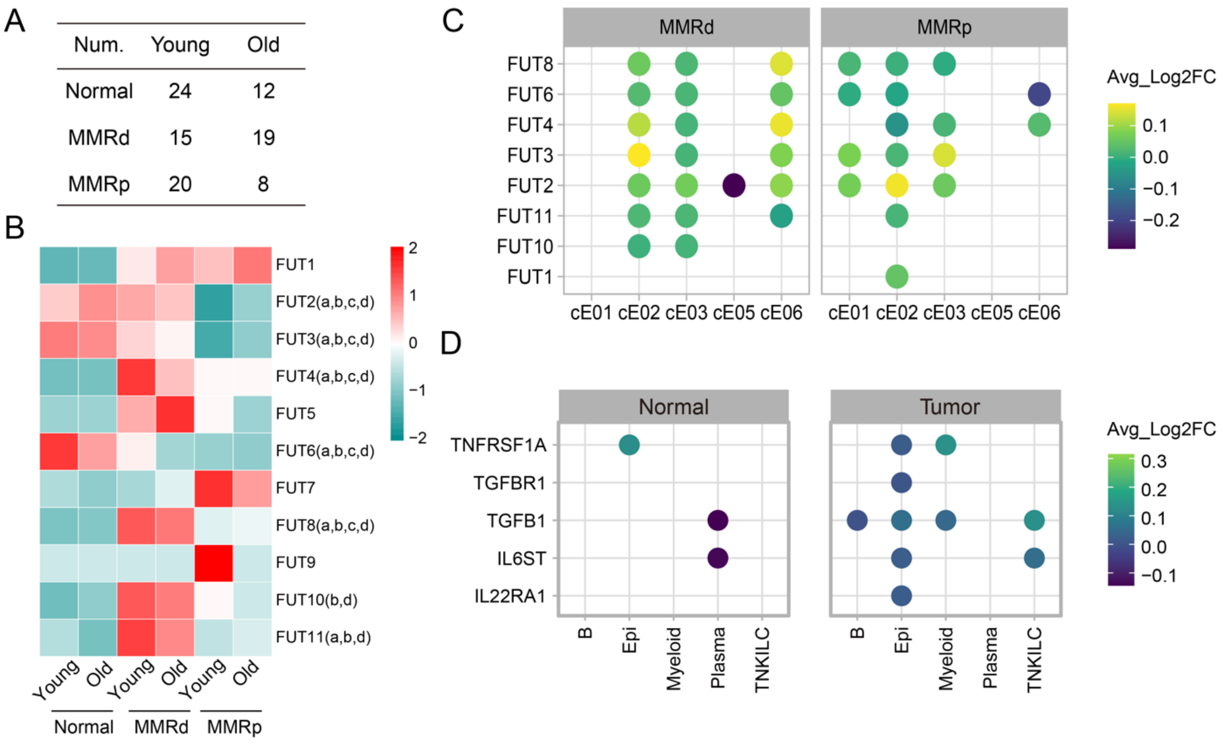

Supplemental Figure 3. Cohort and gene expression in normal and CRC patients. (**A**) Number of samples available for each group. (**B**) Heatmap showing fucosylation genes in normal and tumor patients, a: significant difference between MMRp and MMRd in young patients; b: significant difference between MMRp and MMRd in old patients; c: significant difference between old and young patients with MMRp tumors; d: significant difference between old and young patients with MMRd tumors. (**C** and **D**) Dotplot showed the expression pattern of fucosylation genes among cell types (**C**) and the significant differential genes (padj<0.05) between young and old across all cell types among normal and tumor patients (**D**). The color legend showed the average log2FC between old and young patients.

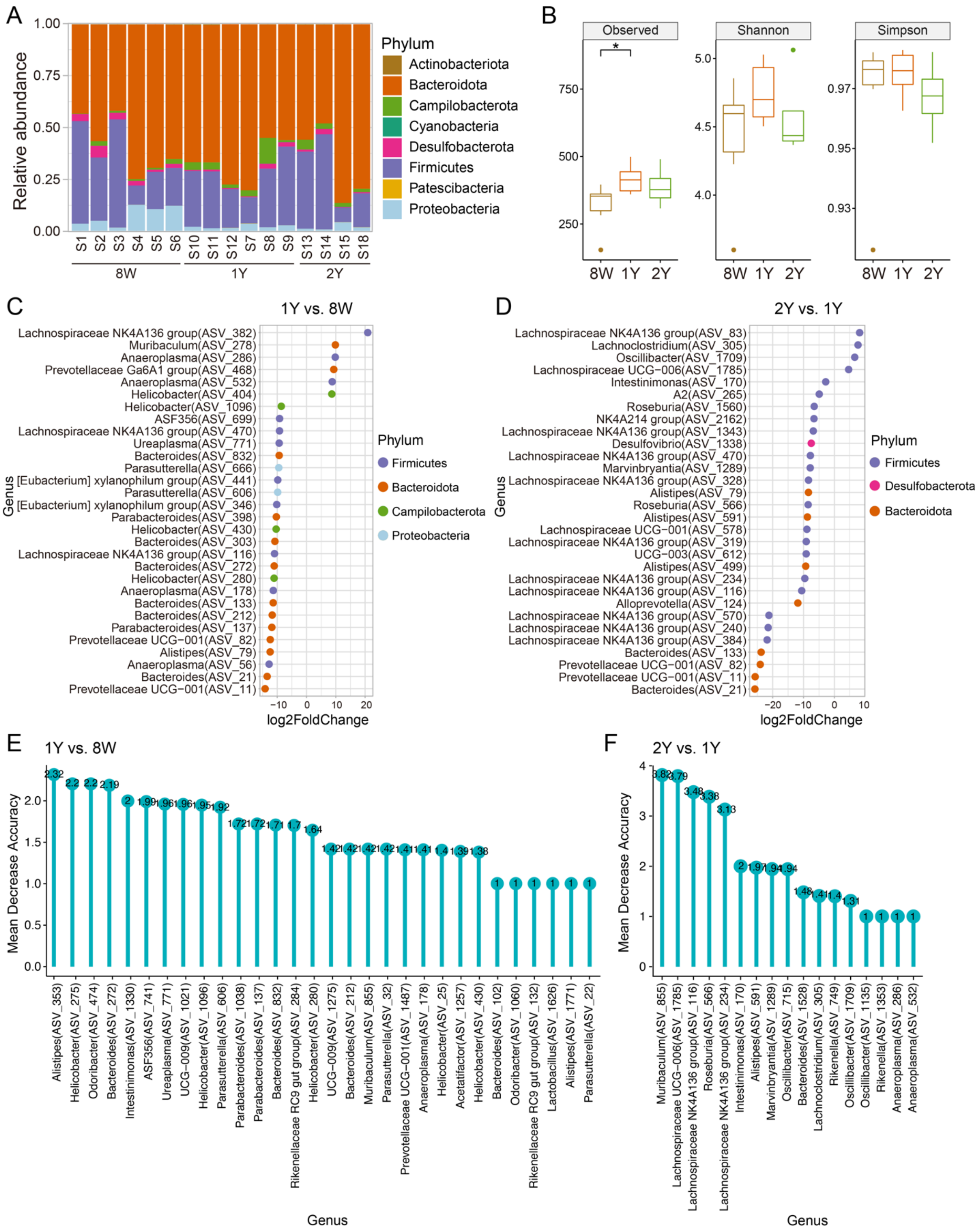

Supplemental Figure 4. Analysis of the diversity and difference of microbiota in 8W, 1Y and 2Y mice. (**A**) Relative abundance of the most prevalent bacterial phyla among all samples.

Color stands for the phylum level. (**B**) Alpha diversity metrics to measure the richness (Observed, Shannon and Simpson) in three groups. T-test was performed between the comparison. (**C** and **D**) Comparison of the significant differential microbiome at the genus level. Only bacteria with significant differences (p-value < 0.05 & $|\log2\text{Fold Change}|>1$) between the 1Y versus 8W (**C**) and 2Y versus 1Y (**D**) are depicted. Colors stand for phylum level. (**E** and **F**) The top 30 differential bacteria distinguish 1Y from 8W (**E**) and 2Y from 1Y (**F**) based on the random forest model. The bar lengths represent mean decrease accuracy, indicating the importance of classification.

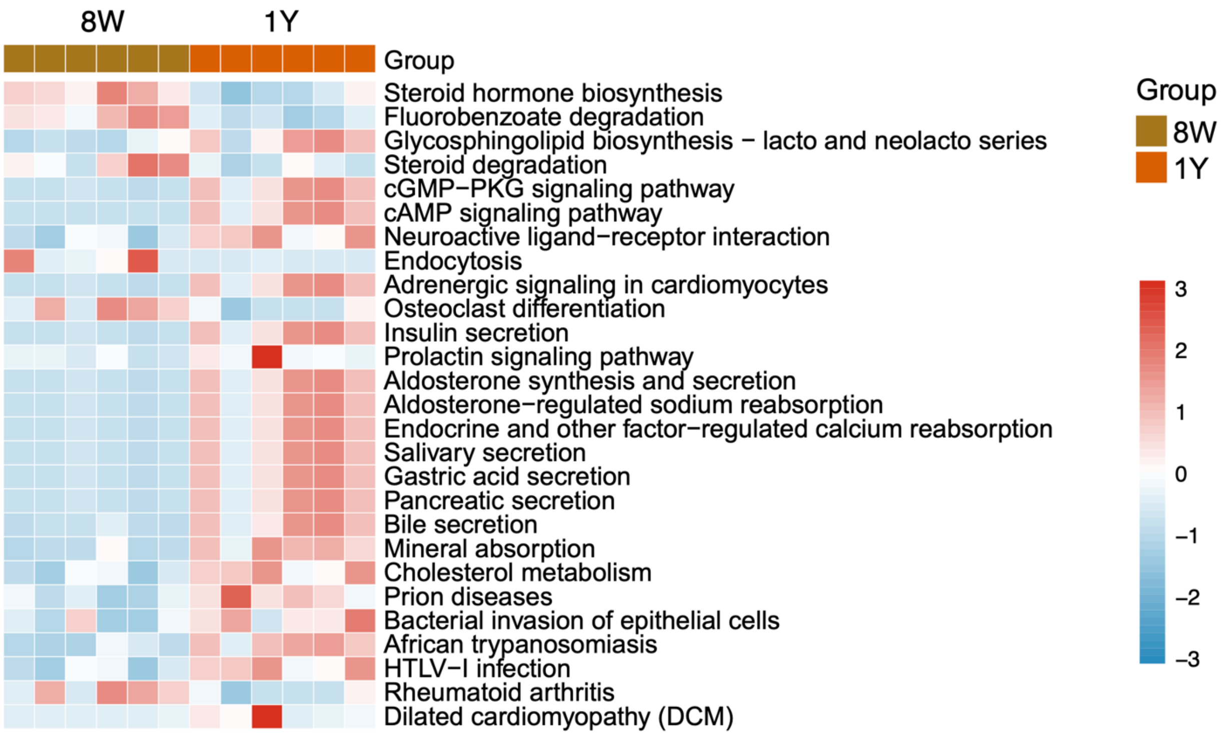

Supplemental Figure 5. Difference of pathways in Stool Samples 8W and 1Y mice.

Representative heatmap of significant KEGG pathways associated with relative bacterial abundance in 1Y versus 8W. The values are scaled by rows (n=6 per group). Only the pathways with significant differences (p-value < 0.05) are shown.

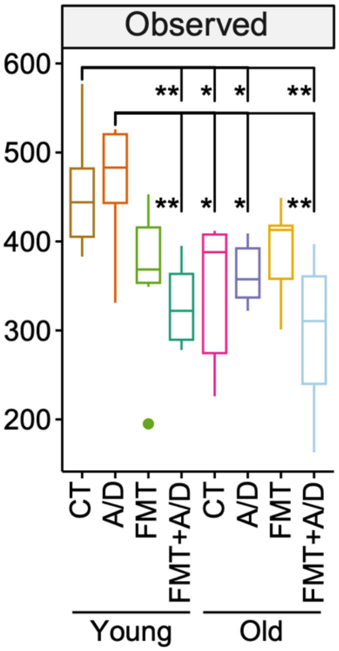

Supplemental Figure 6. Analysis of the alpha-diversity in young and old CRC mice received FMT.

Supplemental Table 1. Clinicopathological characteristics of colon adenocarcinoma tissue microarray (TMA) subjects and samples.

Supplemental Table 2. Detailed clinicopathological information of colon adenocarcinoma tissue microarray (TMA) subjects.

Supplemental Table 3. Quantification of UEA1 intensity in colon adenocarcinoma tissue microarray (TMA) samples.
